# Visualizing inverted sinonasal papilloma through multimodal imaging

**DOI:** 10.11604/pamj.2024.47.128.42976

**Published:** 2024-03-22

**Authors:** Suhit Naseri, Samarth Shukla

**Affiliations:** 1 Department of Pathology, Jawaharlal Nehru Medical College, Sawangi, Meghe, Wardha, Maharashtra, India

**Keywords:** Cutaneous squamous papilloma, nonintestinal type sinonasal adenocarcinoma, nonkeratinizing squamous cell carcinoma

## Image in medicine

Inverted sinonasal papilloma (ISP) is a rare, benign tumor that originates from the Schneiderian membrane lining of the sinonasal tract. Although it is a benign neoplasm, ISP can recur locally and potentially progress to malignancy. Inverted sinonasal papilloma (ISP) is most commonly found in males aged 40-70 years and accounts for about 0.5-4% of all sinonasal tumors. A 72-year-old male with right nasal obstruction underwent a CT scan, which revealed a heterogeneously enhancing soft tissue density lesion in the right nasal cavity (A). A nasal endoscopy further revealed a gray mass in the right nasal cavity with slough and mucopurulent discharge between the lateral wall and septum (B). Grossly, pink-tan-gray, soft to moderately firm polypoid growth is seen (C). Histologically, it is characterized by an endophytic growth pattern, with the tumor growing inward into the underlying stroma (D). The tumor is composed of a mixture of squamous and respiratory epithelium, often with areas of metaplasia, dysplasia, and carcinoma in situ. The stroma is fibrous, with a variable inflammatory infiltrate. Immunohistochemically, the tumor cells express markers of squamous differentiation. The exact cause of ISP is not fully understood, but chronic inflammation, exposure to Human papillomavirus, and genetic alterations are thought to play a role. The treatment of ISP involves complete surgical removal, followed by close clinical and radiological follow-up to monitor for malignant transformation. In conclusion, ISP is a rare benign tumor that can potentially recur or progress to malignancy. Early diagnosis and complete surgical removal are important for the management of ISP.

**Figure 1:**
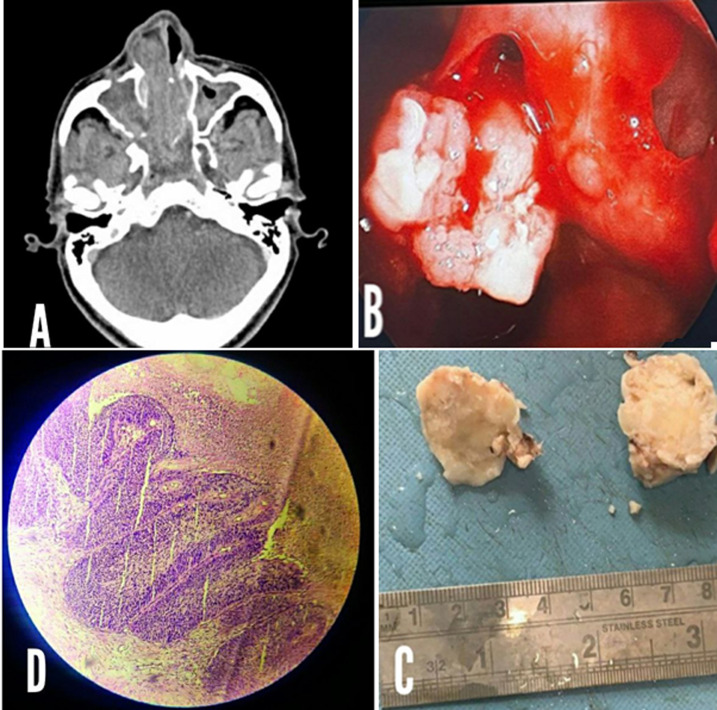
A) computerised tomography; B) endoscopy; C) gross image of the lesion; D) microscopy of the lesion

